# Supplementary figures and images for: Increased expression of SPRR1A is associated with a poor prognosis in pancreatic ductal adenocarcinoma
Source: PLoS One. 2022 May 26;17(5):e0266620. doi: 10.1371/journal.pone.0266620 (PMC9135243; doi:10.1371/journal.pone.0266620)

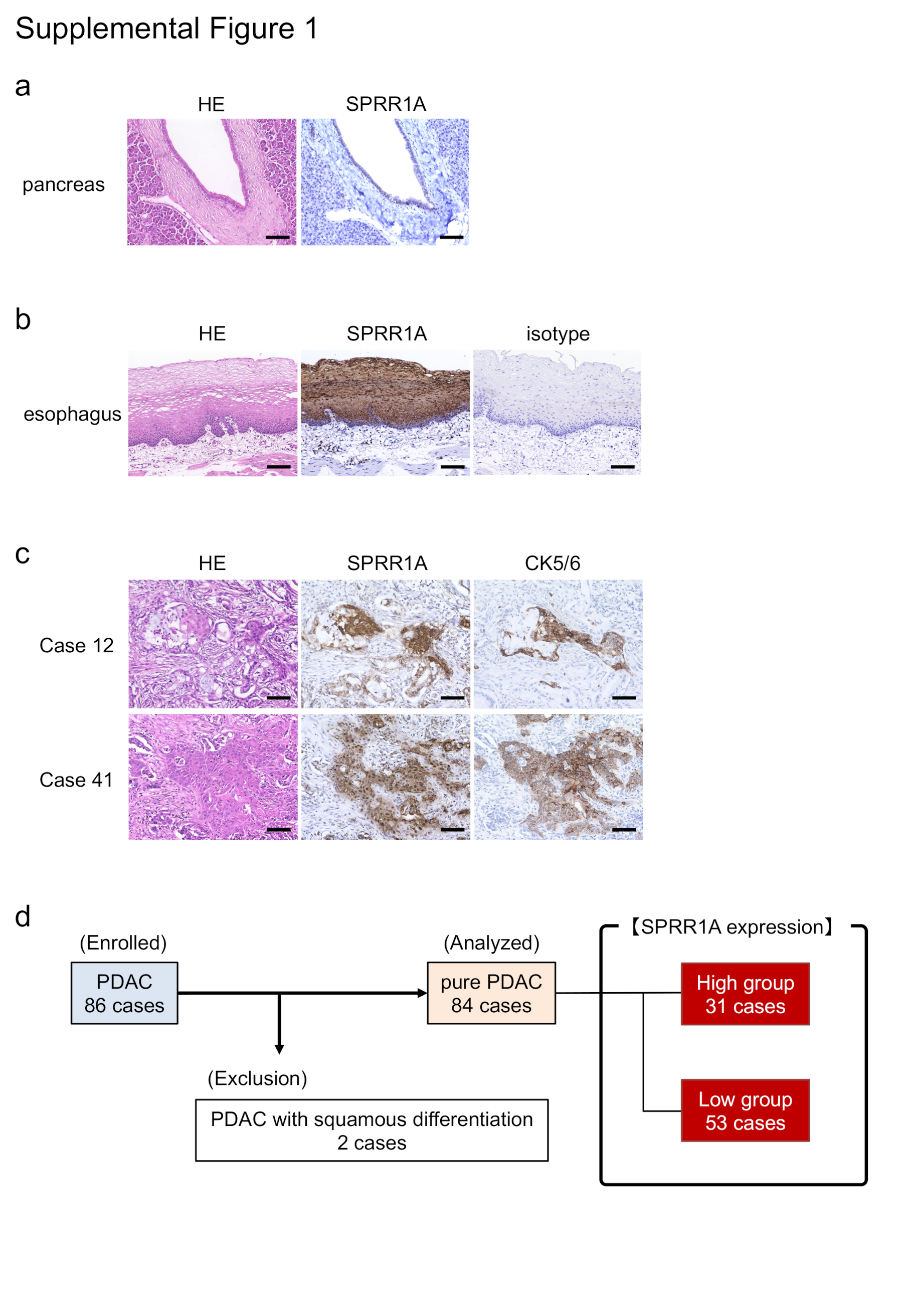

Supplement: S1 Fig — (a) The expression of SPRR1A in the normal pancreatic ductal epithelium. Scale bars, 500 μm. (b) The expression of SPRR1A in the normal esophageal epithelium. Scale bars, 500 μm. (c) The expression of SPRR1A and CK5/6 in two PDAC cases with squamous differentiation (Case 12 and 41). Scale bars, 500 μm. (d) A flowchart of the case selection in the current study. (TIF) [file pone.0266620.s001.tif]

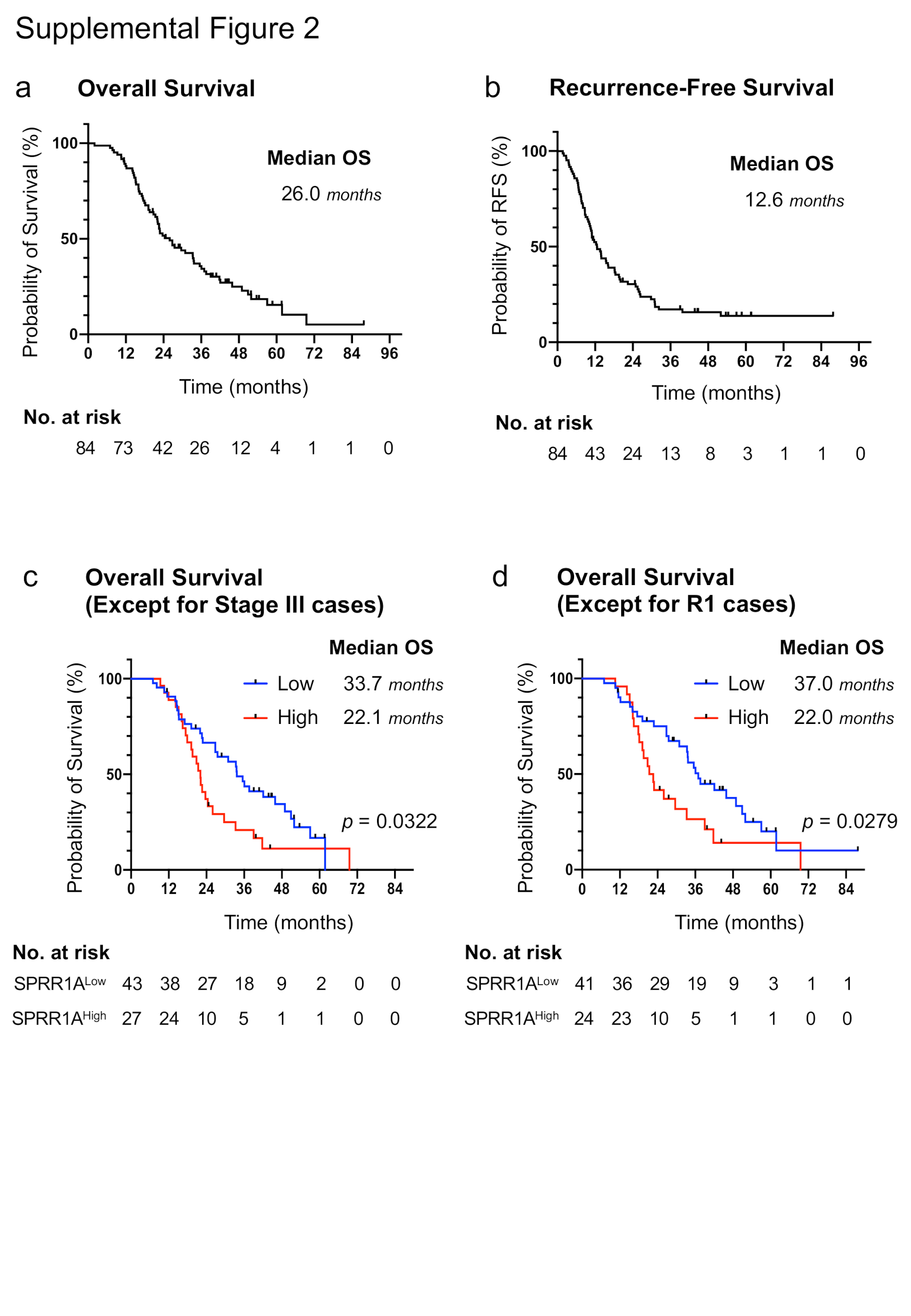

Supplement: S2 Fig — (a) Kaplan-Meier estimates of the OS in all PDAC cases. (b) Kaplan-Meier estimates of the RFS in all PDAC cases. Tick marks indicate censored data. (c) Kaplan-Meier estimates of the OS stratified by the SPRR1A expression in PDAC cases, except for those with stage III disease. (d) Kaplan-Meier estimates of the OS stratified by the SPRR1A expression in PDAC cases, except for those with R1. (TIF) [file pone.0266620.s002.tif]

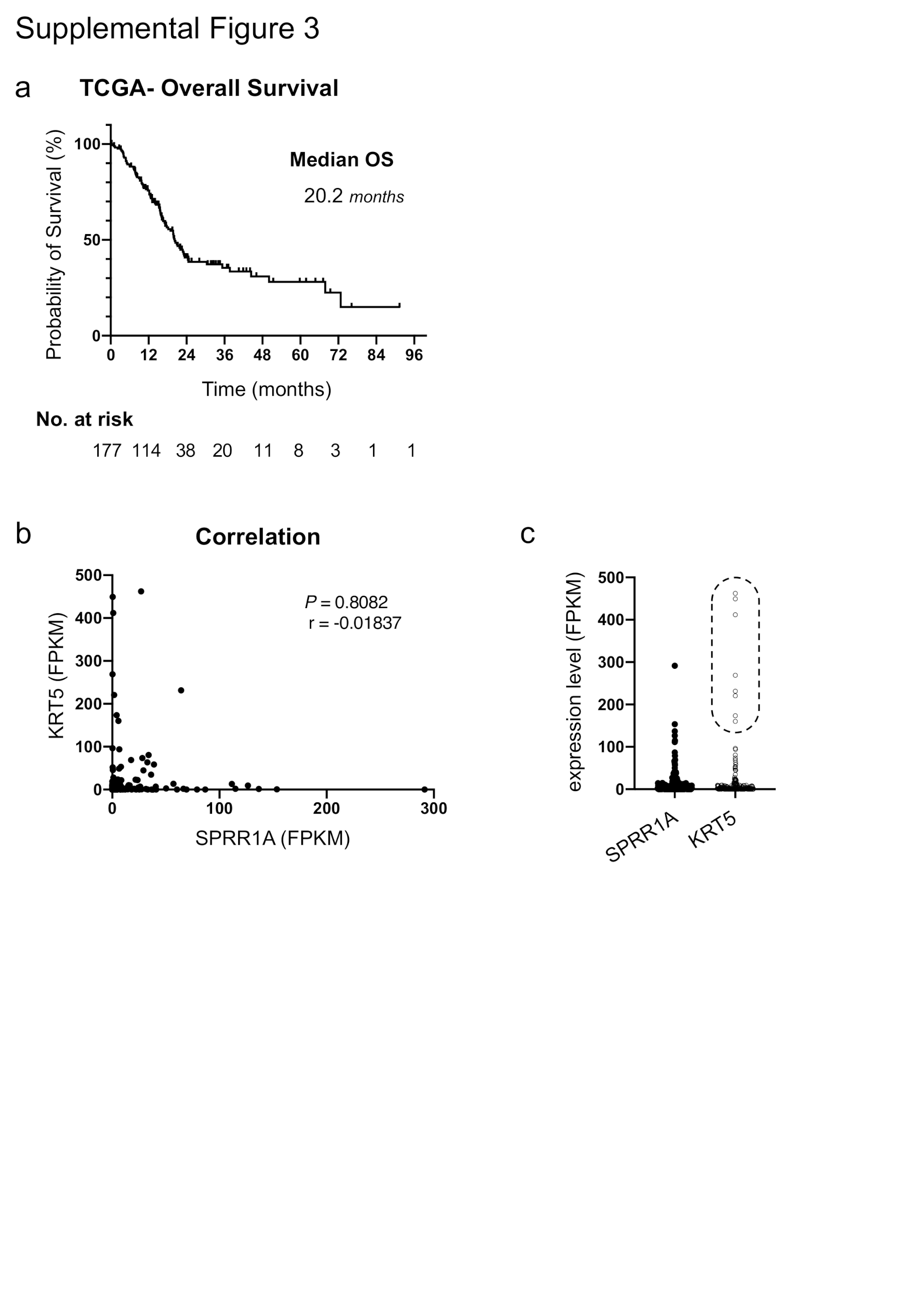

Supplement: S3 Fig — (a) Kaplan-Meier estimates of the OS in all cases from the TCGA-PAAD data. (b) Correlation between the transcript level of SPRR1A and KRT5. Correlation analyses were performed using Pearson’s product-moment correlation coefficient. (c) The transcript level of SPRR1A and KRT5. The dotted line shows the cases with a high transcript level of KRT5 (above the average plus two s.d.). (TIF) [file pone.0266620.s003.tif]

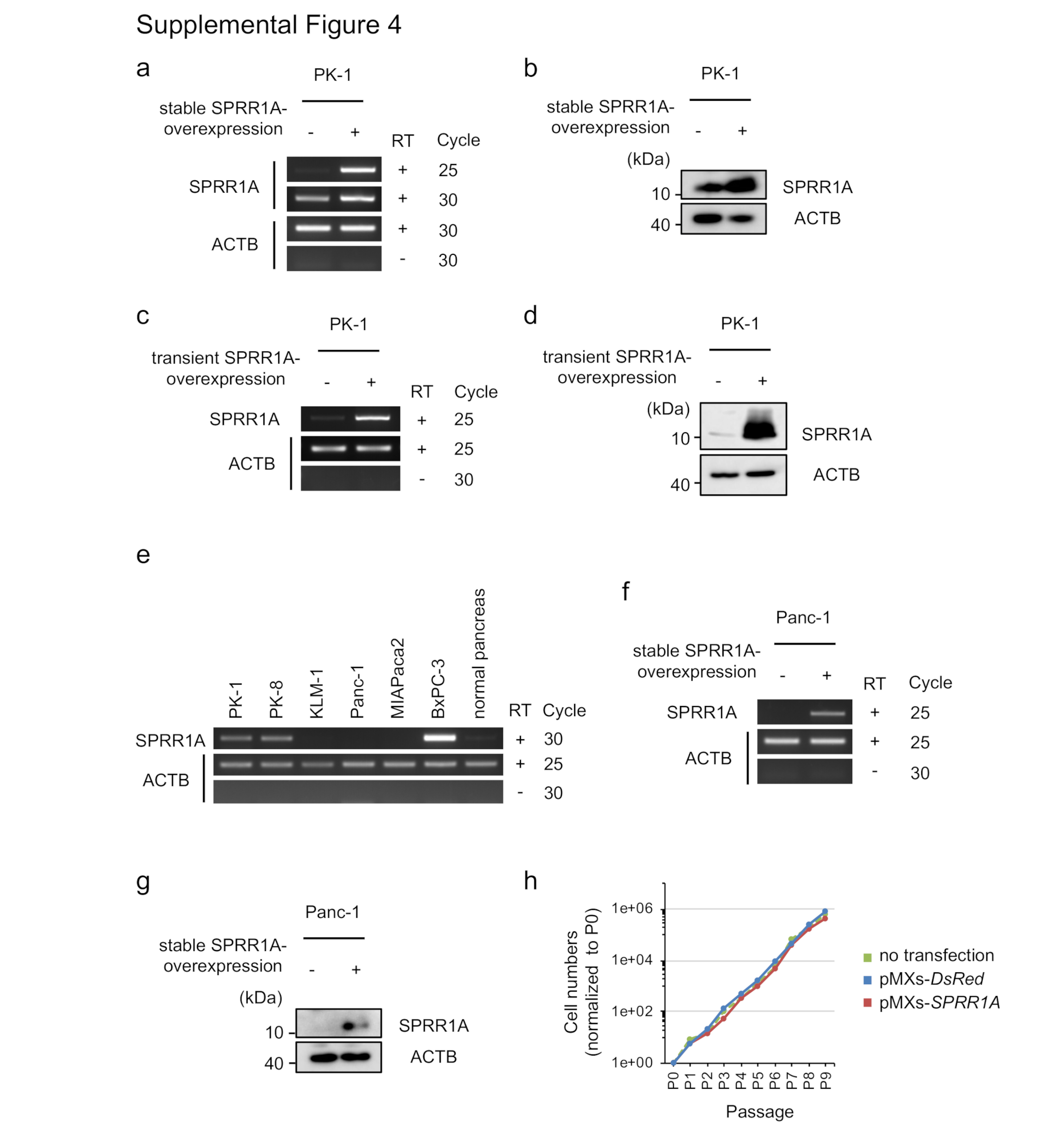

Supplement: S4 Fig — (a) The mRNA expression of SPRR1A in stable SPRR1A-overexpressing cells derived from PK-1 was examined by semi-quantitative RT-PCR using total SPRR1A primers. (b) The protein expression of SPRR1A in stable SPRR1A-overexpressing cells derived from PK-1 was examined by Western blotting. (c) The mRNA expression of SPRR1A in transient SPRR1A-overexpressing cells derived from PK-1 was examined by semi-quantitative RT-PCR using total SPRR1A primers. (d) The protein expression of SPRR1A in transient SPRR1A-overexpressing cells derived from PK-1 was examined by Western blotting. (e) The mRNA expression of SPRR1A in various pancreatic cancer cell lines and normal pancreas tissue was examined by semi-quantitative RT-PCR using endogenous SPRR1A primers. (f) The mRNA expression of SPRR1A in stable SPRR1A-overexpressing cells derived from Panc-1 was examined by semi-quantitative RT-PCR using total SPRR1A primers. (g) The protein expression of SPRR1A in stable SPRR1A-overexpressing cells derived from Panc-1 was examined by Western blotting. (h) Cell proliferation. The cell number of SPRR1A-transduced Panc-1 was counted every three to four days after transduction. RT- indicates control PCR without reverse transcription. (TIF) [file pone.0266620.s004.tif]

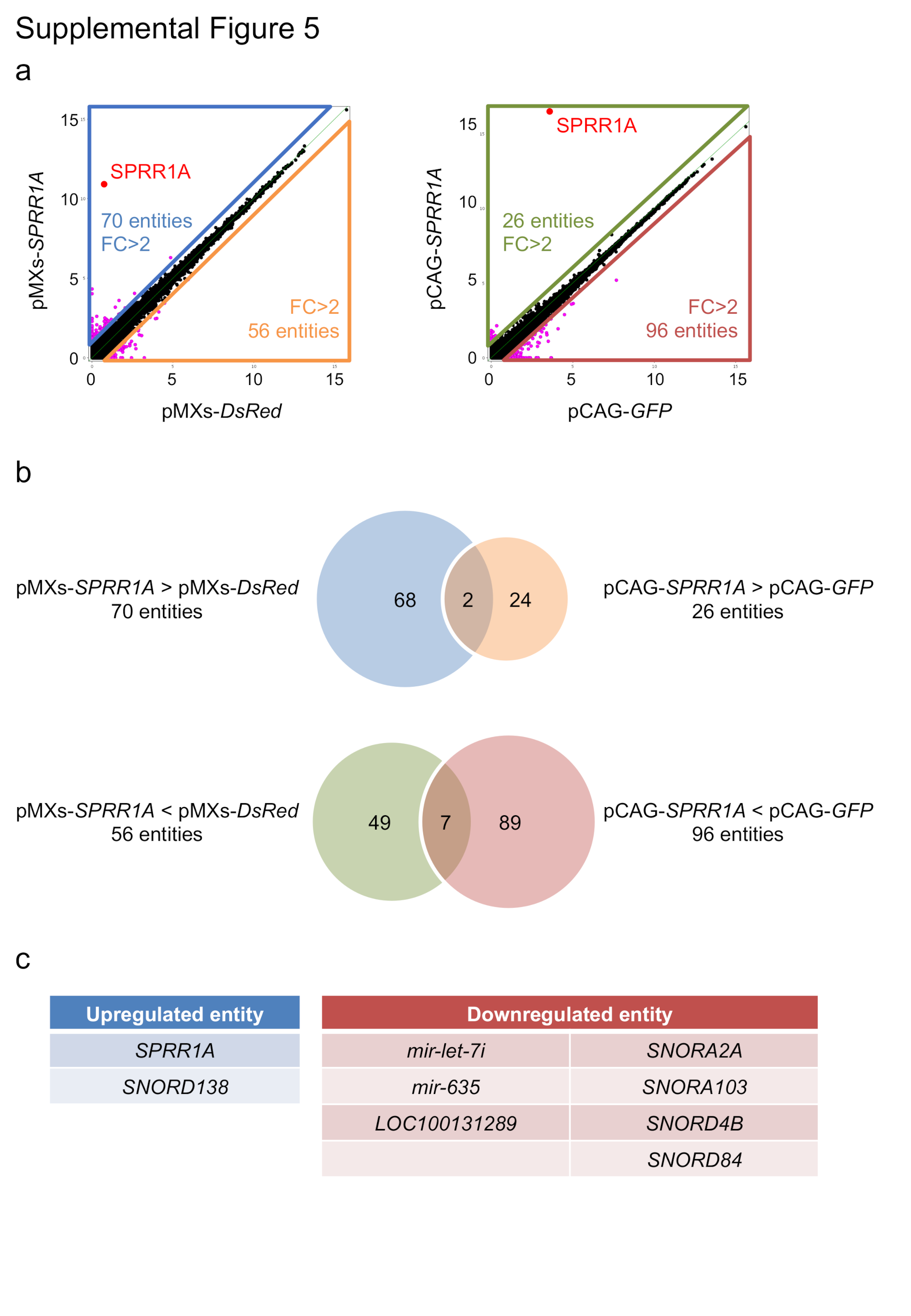

Supplement: S5 Fig — (a) Seventy upregulated and 26 downregulated entities were identified with a more than two-fold change in expression by stable SPRR1A overexpression (left). Fifty-six upregulated and 96 downregulated entities were identified with a more than two-fold change in expression by transient SPRR1A overexpression (right). Magenta dots show entities with more than two-fold changes. The scale is shown in base two logarithm. (b) Venn diagrams of the entities more highly expressed in stable and transient overexpression cells than in controls (above). Venn diagrams of the entities whose expression was downregulated in stable and transient overexpression cells than in controls (below). (TIF) [file pone.0266620.s005.tif]

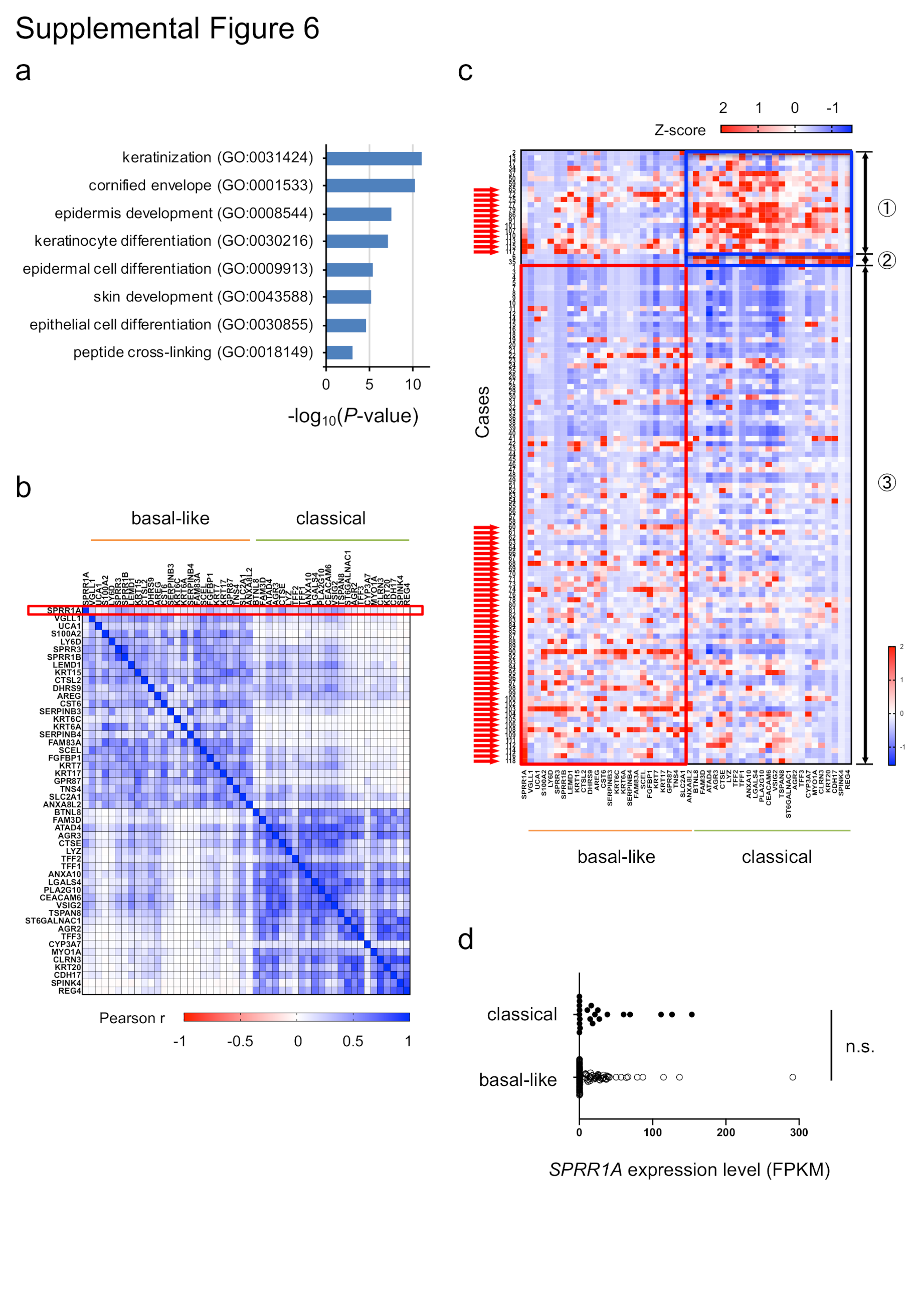

Supplement: S6 Fig — (a) The blue bars indicate GO terms (p < 0.001) related to the upregulated entities in the high-SPRR1A-expression group in TCGA-PAAD data. (b) The heatmap indicates the result of correlation analyses between SPRR1A and the signature genes of the molecular subtypes of PDAC in TCGA-PAAD cases. (c) The heatmap indicates the expression of SPRR1A and the signature genes of the molecular subtypes of PDAC, classified by K-means cluster analyses in TCGA-PAAD cases. The circled numbers 1, 2, and 3 indicate clusters, respectively. Clusters 1 and 2 represent the "classical" subtype, while cluster 3 represents the "basal-like" subtype. Arrows indicate cases with a high SPRR1A expression. (d) The comparison of the SPRR1A expression (FPKM) between the "basal-like" (n = 96) and "classical" (n = 22) subtypes indicated in S6C Fig. n.s., not significant, unpaired t-test. (TIF) [file pone.0266620.s006.tif]

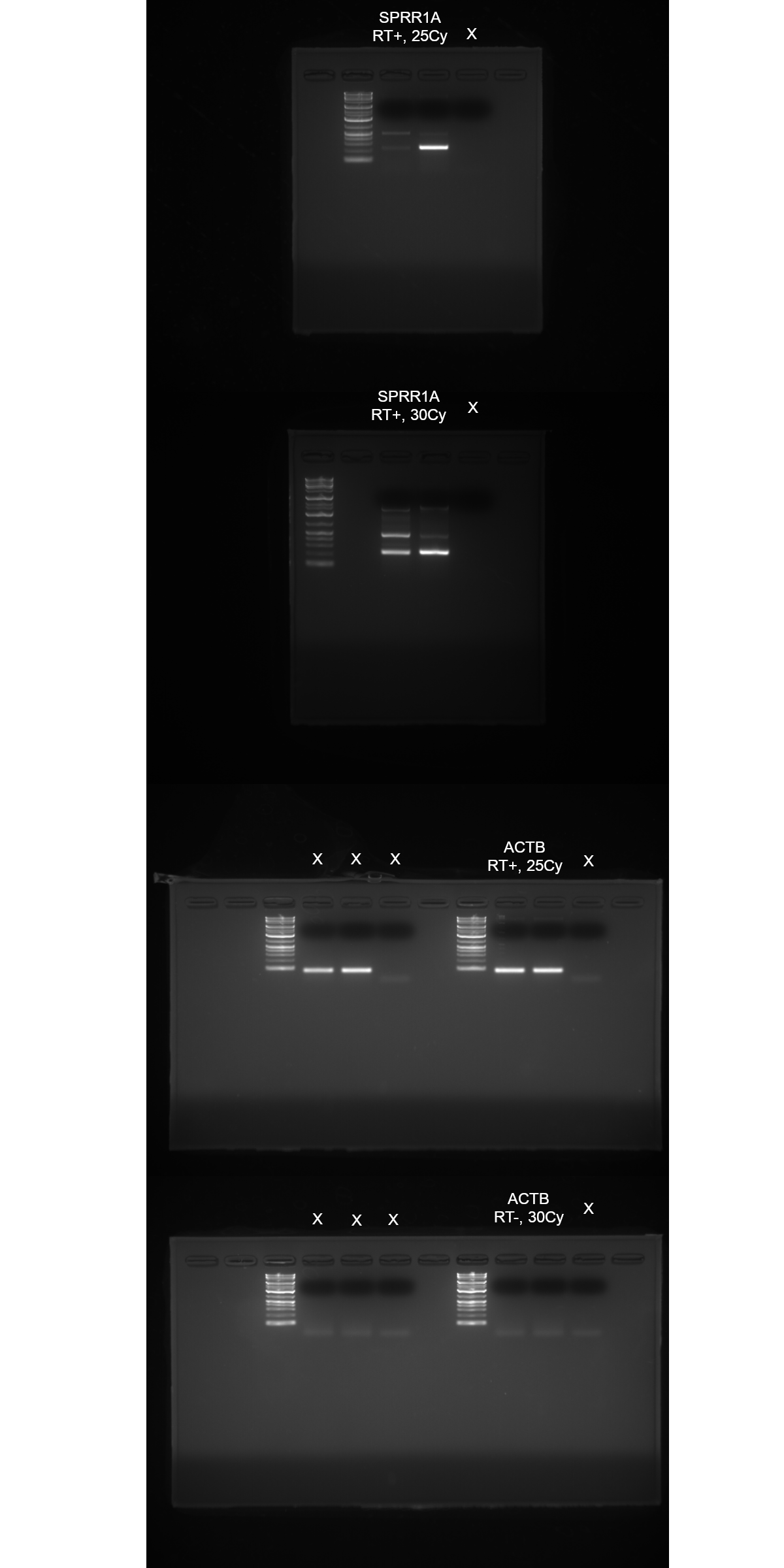

Supplement: S1 File — (ZIP) [file pone.0266620.s012.zip › S-fig.4a.tif]

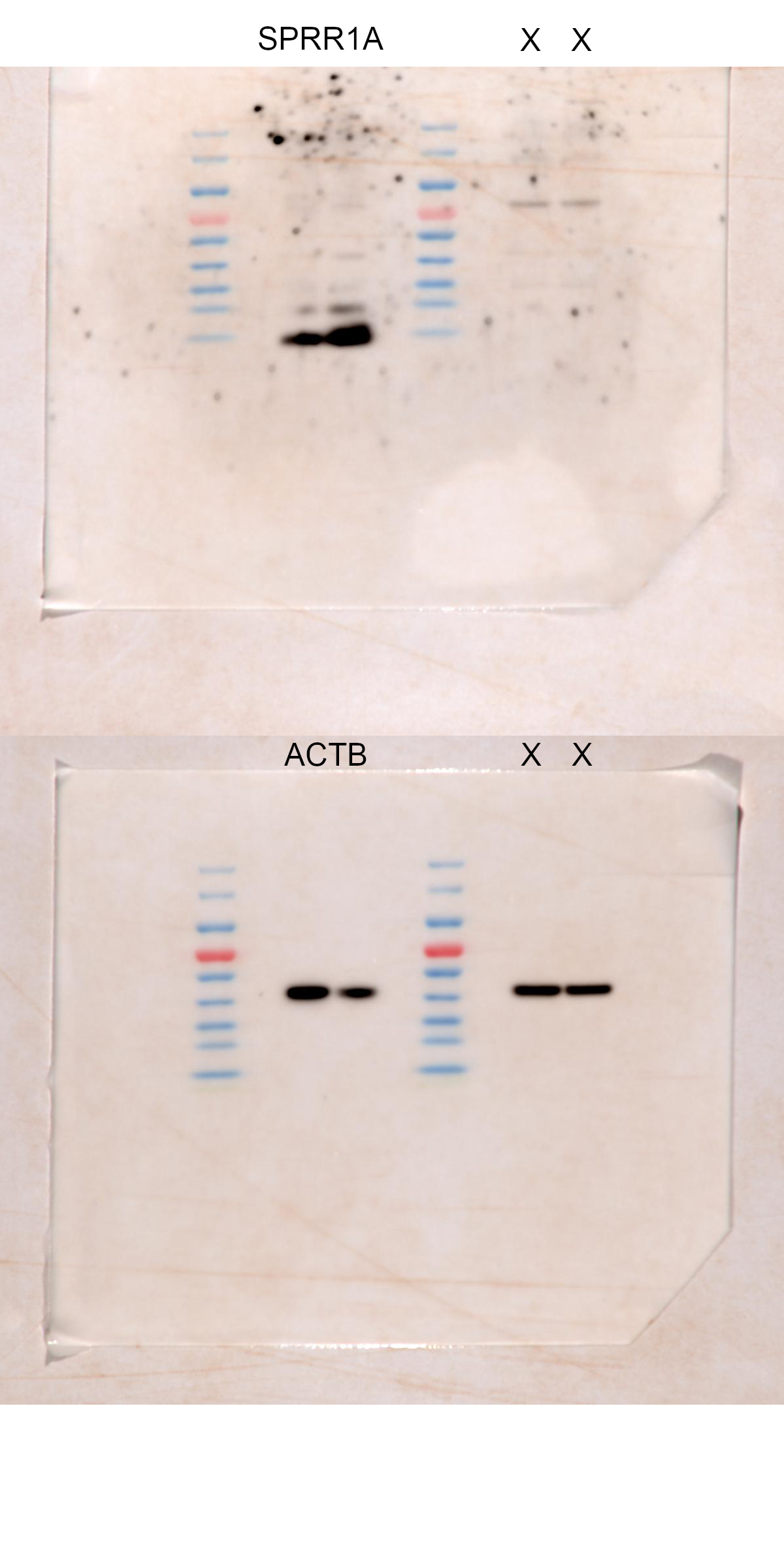

Supplement: S1 File — (ZIP) [file pone.0266620.s012.zip › S-fig.4b.tif]

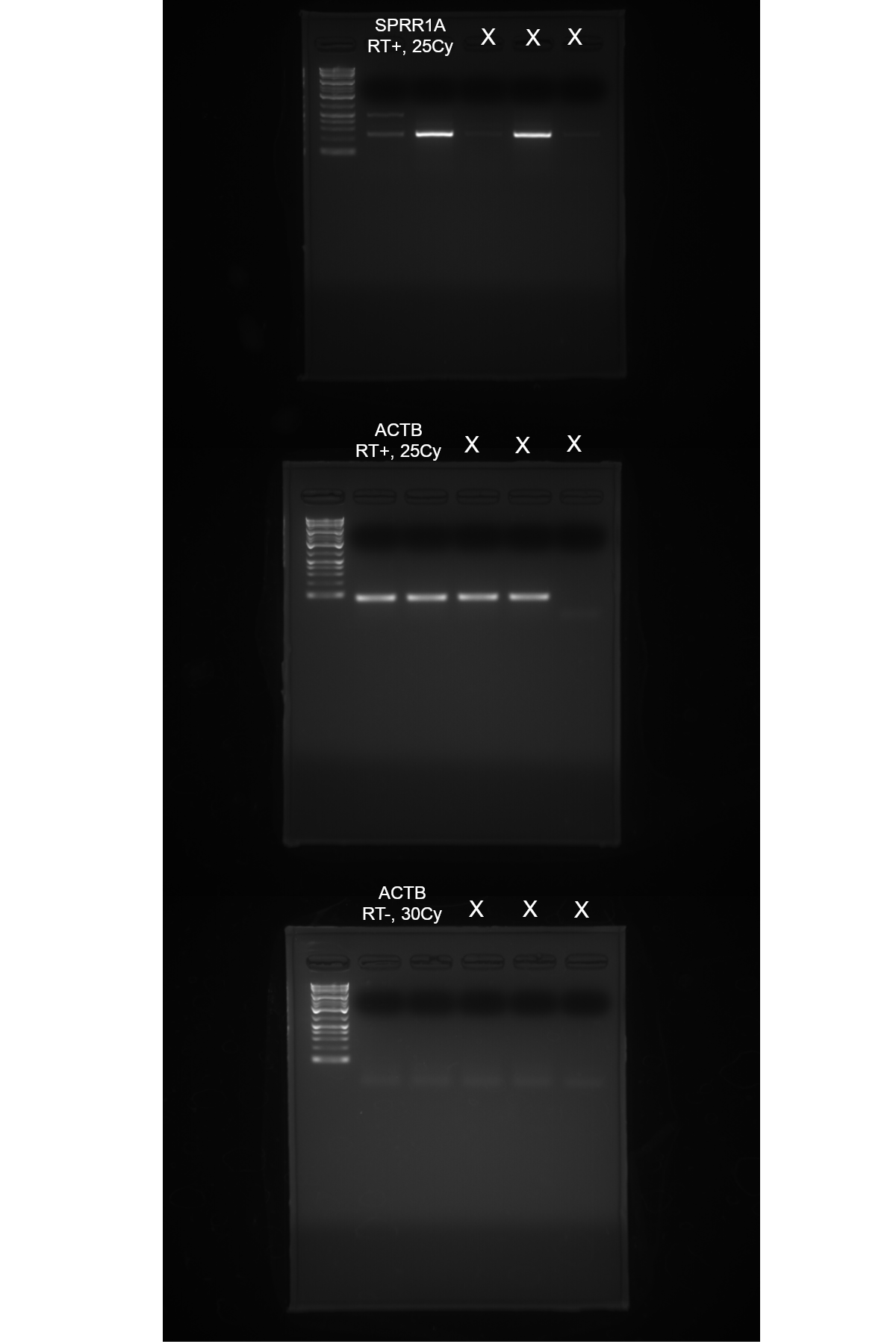

Supplement: S1 File — (ZIP) [file pone.0266620.s012.zip › S-fig.4c.tif]

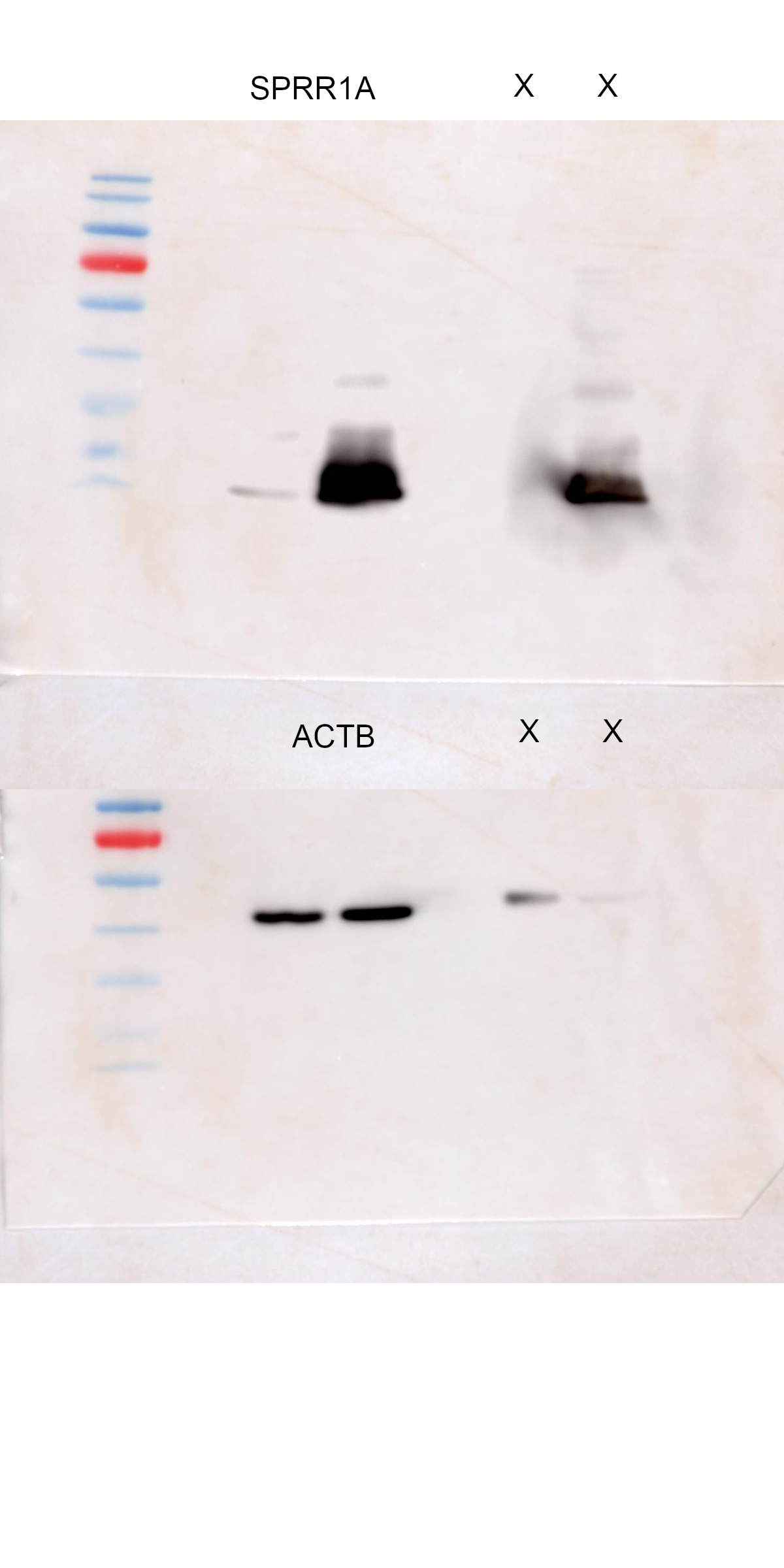

Supplement: S1 File — (ZIP) [file pone.0266620.s012.zip › S-fig.4d.tif]

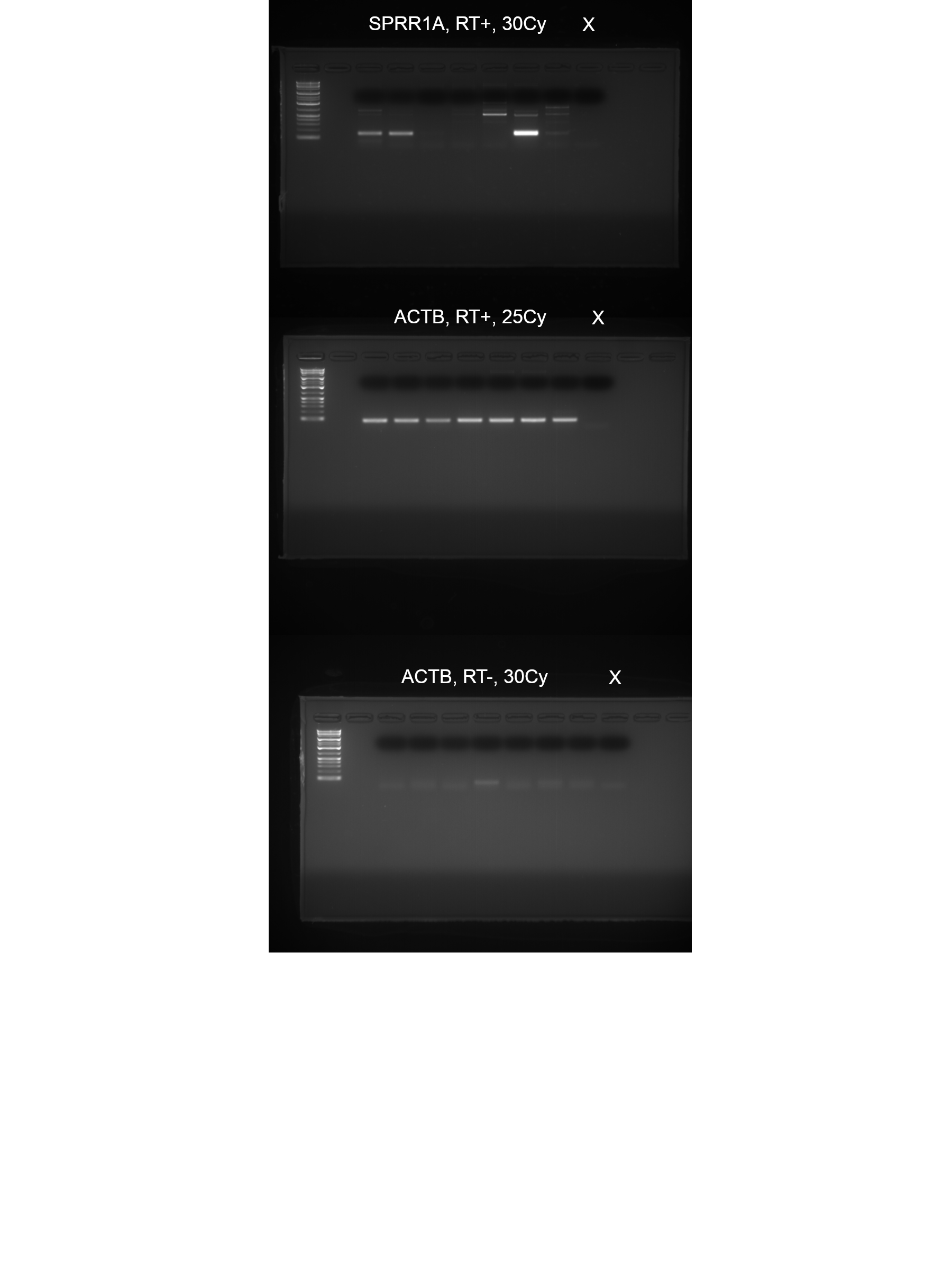

Supplement: S1 File — (ZIP) [file pone.0266620.s012.zip › S-fig.4e.tif]

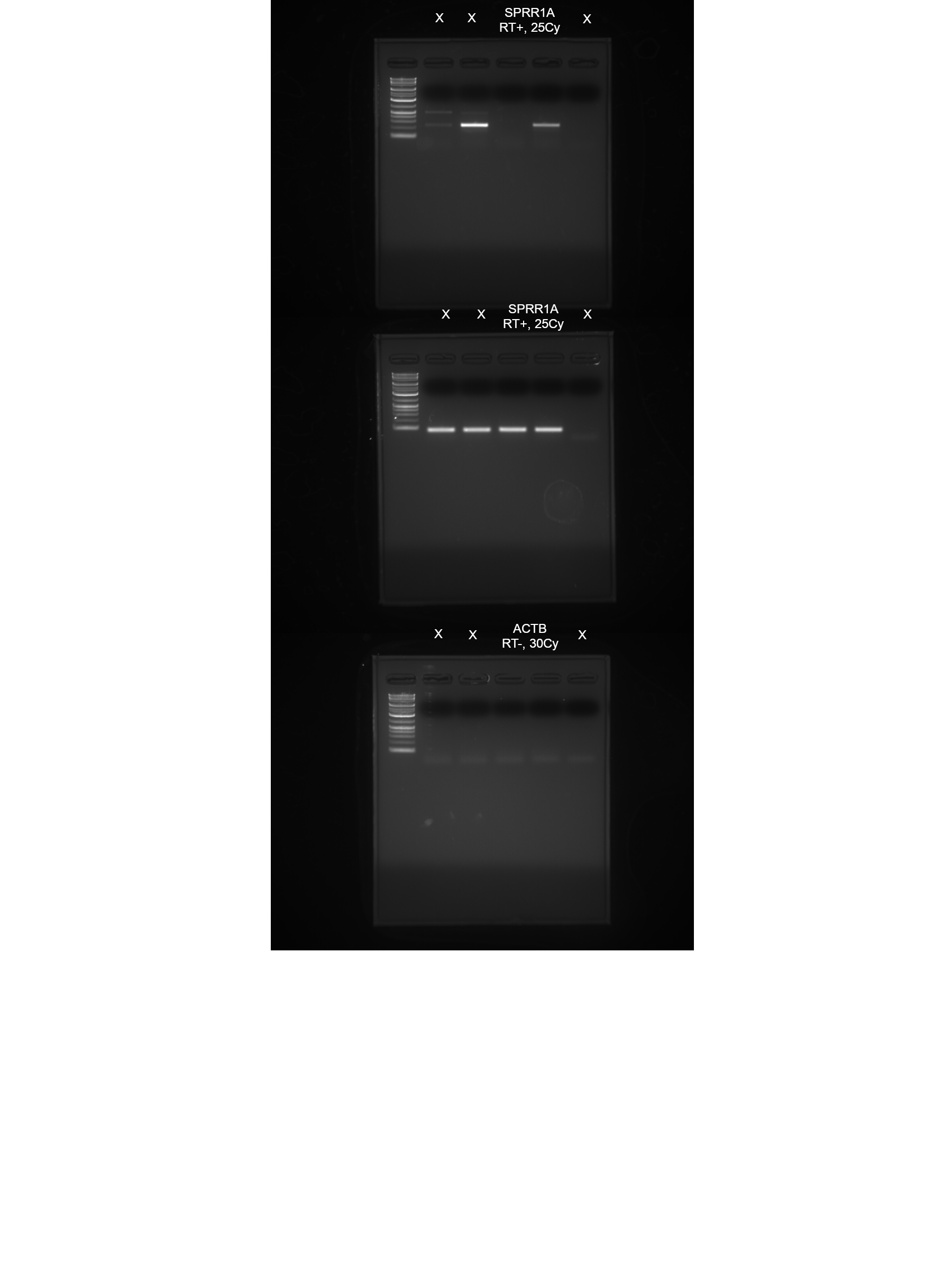

Supplement: S1 File — (ZIP) [file pone.0266620.s012.zip › S-fig.4f.tif]

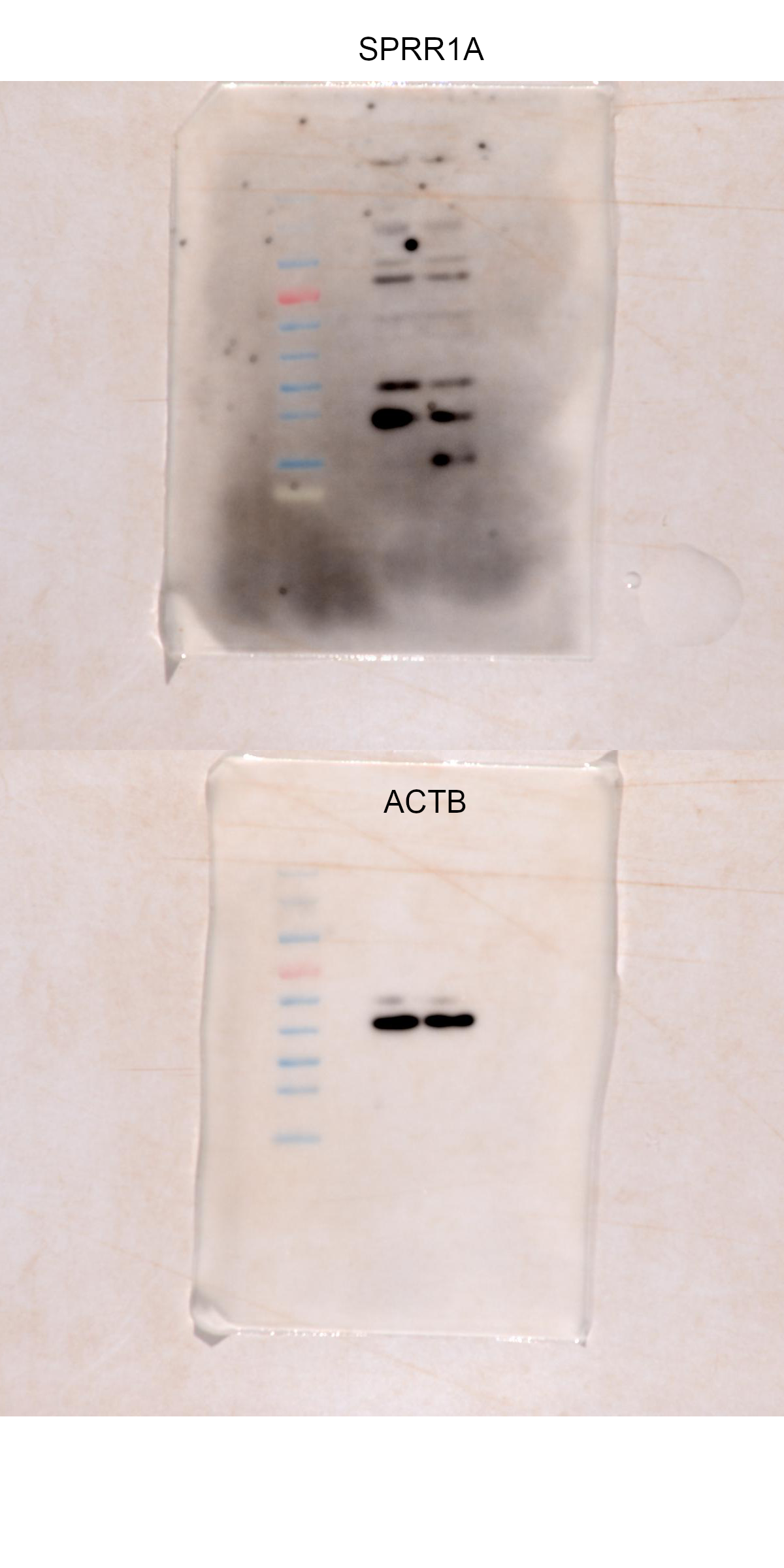

Supplement: S1 File — (ZIP) [file pone.0266620.s012.zip › S-fig.4g.tif]
